# Supplementary material for: Competing effects of vegetation density on sedimentation in deltaic marshes
Source: Nat Commun. 2022 Aug 8;13:4641. doi: 10.1038/s41467-022-32270-8 (PMC9360023; doi:10.1038/s41467-022-32270-8)
Supplement: Supplementary file 1 — Supplementary Information [file 41467_2022_32270_MOESM1_ESM.pdf]

Supplementary Information for

Competing effects of vegetation density on sedimentation in deltaic marshes

Yuan Xu[1,2]*, Christopher R. Esposito[3,4], Maricel Beltrán Burgos[4], Heidi M. Nepf[2]

[1]State Key Laboratory of Hydroscience and Engineering, Hydraulic Engineering, Tsinghua University, Beijing, China

[2]Depart. of Civil Engineering, Massachusetts Institute of Technology, Cambridge, MA, USA

[3]The Water Institute of The Gulf, Baton Rouge, LA, USA

[4]Depart. of Earth and Environmental Sciences, Tulane University, New Orleans, LA, USA

*e-mail: xuyuan18@mails.tsinghua.edu.cn

This PDF file includes:

Sections S1 to S5

Figures S1 to S5

S1. Equilibrium of sedimentation rate

The model separately considered sediment deposition and resuspension, which together yield net deposition (sedimentation). The sedimentation rate q_d (particles/m^2/s) at time t was

$$q_d(t) = R_d(t) - R_r(t) = \frac{M_{nd,n}(t) - M_{nd,n}(t-\Delta T)}{A \cdot \Delta T}, \qquad \text{(S1)}$$

in which R_d is the deposition rate, R_r is the resuspension rate, A is the domain area, and ΔT is the time interval. Note that q_d in Eq. (M11) is the time-average of $q_d(t)$ in Eq. (S1). Fig. S1 shows the evolution of $q_d(t)$ normalized by the sediment supply rate q_s for a case with resuspension (n=80 m^{-2}) and without resuspension (n=160 m^{-2}). The sedimentation rates in these cases (and similarly for other cases) reached equilibrium at t ≈200s. For cases without resuspension, once equilibrium was reached the sedimentation rate was equal to the sediment supply rate, i.e., $q_d(t)/q_s$ =1, as expected.

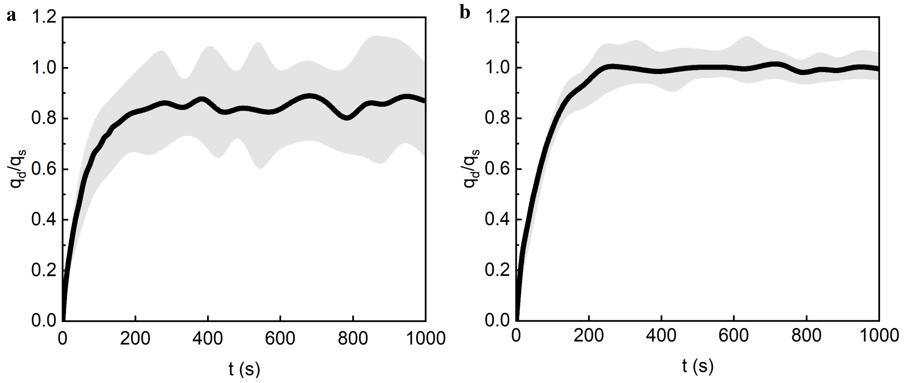

Fig. S1 | The time evolution of net deposition rate normalized by the supply rate (q_d/q_s). a, Case with resuspension (n=80 m^{-2}, d=1.0 cm, S=0.0005). The black line was smoothed with a moving average. The gray area indicates the data range without smoothing and is shown to demonstrate the range of behavior inherent in the stochastic

representation of resuspension. **b,** Case without resuspension (n=160 m^{-2}, d=1.0 cm, S=0.0005).

S2. Suspended sediment concentration profile for different Peclet numbers

The presence of vegetation affects the vertical diffusivity, D_z, which impacts on the shape of suspended sediment concentration (SSC) profile (Fig. S2). The shape of the SSC profile is often related to a Peclet number defined for vertical transport of sediment ($P_e = w_s H/D_z$, with settling velocity w_s, water depth H), which connects the particle settling and vertical diffusion. Specifically, as n increased from 10 to 60 m^{-2}, the velocity and diffusivity decreased, so that the Peclet number increased from 1.4 to 2.6. This indicated that the settling process became more important in sediment transport as stem density increased. Accordingly, the profile of SSC shifted further away from a uniform distribution.

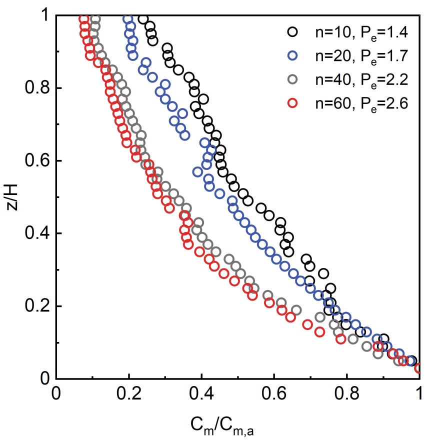

Fig. S2 | Suspended sediment concentration, C_m, normalized by reference $C_{m,a}$ (at z=a=0.05H). As stem density increased (black, blue, grey, red symbols), vertical diffusivity decreased, leading to greater vertical variation in suspended sediment concentration, with P_e=1.4 (n=10 m^{-2}), 1.7 (n=20 m^{-2}), 2.2 (n=40 m^{-2}), and 2.6 (n=60 m^{-2}).

S3. Length-scales of sedimentation patterns

Two patterns for the spatial distribution in sedimentation emerged from the model, depending on whether, or not, resuspension occurred (Fig. 4). Each pattern can be described by a length-scale. In cases without resuspension (Pattern 1), all particles supplied to the marsh deposited within a finite distance from the marsh edge, L_{nd}, called the length scale of sedimentation. L_{nd} was defined by the distance at which deposition declined to 5% of the maximum sedimentation (i.e., $M_{nd,1m}$ at x=1m). For cases without resuspension, the sediment Peclet number was large enough that sediment transport was dominated by gravity (settling). For these conditions, previous studies have shown that the settling time-scale, H/w_s, defines the sedimentation distance, UH/w_s[1]. Fig. S3a shows that $L_{nd} = (2.1 \pm 0.1)\ UH/w_s$.

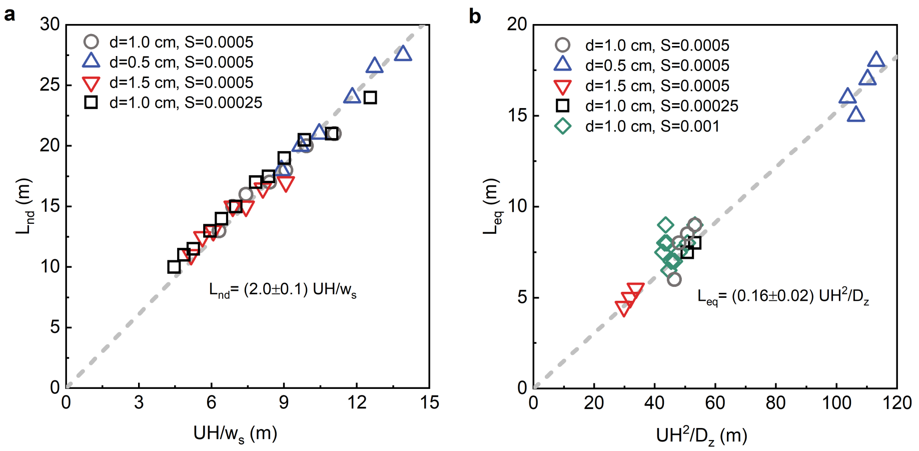

Fig. S3 | Length-scales of sedimentation patterns. a, When resuspension was absent (Pattern 1), sedimentation distance L_{nd} was linearly correlated with UH/w_s. Gray dashed line indicates $L_{nd} = 2.0\,UH/w_s$. Note that cases with S=0.001 were excluded, because resuspension occurred for all densities. The P_e ranged from 3.9 to 9.0. **b,** When resuspension was present (Pattern 2) sedimentation equilibrium distance L_{nd} varied linearly with UH^2/D_z. Gray dashed line indicates $L_{eq} = 0.16\,UH^2/D_z$. The Peclet number in **b** ranged from 0.6 to 3.0.

When stem density is small, the presence of resuspension remobilizes deposited sediments, allowing them to be carried farther into the marsh (Pattern 2). The sedimentation initially increases with distance from the marsh edge (x), then reaches a constant once the profile of SSC reaches equilibrium. The equilibrium distance L_{eq} was defined by the distance at which the sedimentation increased to 95% of the final value. For these conditions, the Peclet number is small and diffusion dominates transport, such that the time-scale needed to reach the equilibrium SSC profile, $T_{eq} = H^2/D_z$, is related to diffusivity and water depth[1-3]. The equilibrium distance L_{eq} is related T_{eq}. Specifically, $L_{eq} = (0.16 \pm 0.02)\ UH^2/D_z$ (Fig. S3b). Cases falling farther from the fitted curve (e.g., green diamonds) have larger values of Peclet number, such that gravity (settling) also influences T_{eq}.

S4. Spatial evolution of SSC profile

The suspended sediment is uniformly distributed over depth as it enters the marsh (x=0 in Fig. S4), and the vertical profile of SSC subsequently evolves as water moves into the marsh, adjusting to the magnitude of vertical diffusivity within the vegetation. Specifically, the SSC profile becomes increasingly non-uniform, with relatively higher concentration near the bed. The simulated concentration profiles at x=10 m to 20 m differ by less than 5%, indicating that the SSC has reached the equilibrium profile. This distance defines L_{eq}. As the SSC profile evolves from the marsh edge, the near-bed concentration gradually increases, which produces the increasing magnitude of sedimentation between the marsh edge and distance L_{eq}(Fig. 4b).

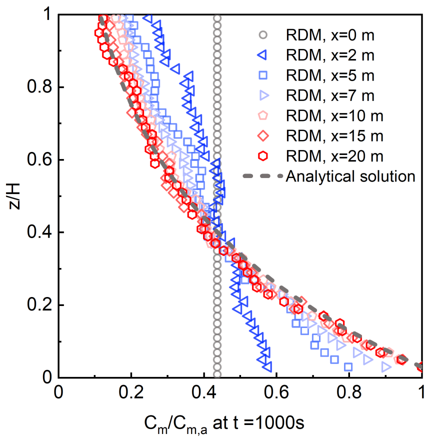

Fig. S4 | Evolution of SSC profile with distance from marsh edge for n=40 m^{-2}, d=1.0 cm, S=0.0005. Vertical profile of normalized SSC ($C_m/C_{m,a}$) at x = 0 to 20 m, with $C_{m,a}$ the SSC at z=a=0.05H. Grey to blue to red colors represent increasing distance. Gray dashed line indicates the analytical solution (Eq. (S3)).

Once the vertical profile of SSC reaches equilibrium (Fig. S4), the profile shape is described the balance of the vertical advection and diffusion,

$$D_z \frac{dC_m}{dz} + w_s C_m = 0 \qquad \text{(S2)}$$

Within a canopy of rigid, emergent cylinders, k_t and l_t are uniform over vertical distance z, leading to a uniform distribution of D_z. With constant D_z and w_s, Eq. (S2) yields[4]

$$\frac{C_m}{C_{m,a}} = \exp\left[-\frac{w_s}{D_z}(z-a)\right] \qquad \text{(S3)}$$

in which, a is the reference height, taken as $a = 0.05H$. The SSC profile estimated from Eq. (S3) (gray dashed line in Fig. S4) has good agreement with the RDM simulation.

S5. Supplement to Figure 5: Modeling curves for each measurement condition

Each measurement conditions reported in Table 1 occurred under a different water slope, which was associated with a different modeled curve of $\widetilde{q_d}$ versus vegetation density (leaves/m^2). The individual curves are shown, labeled with the slope.

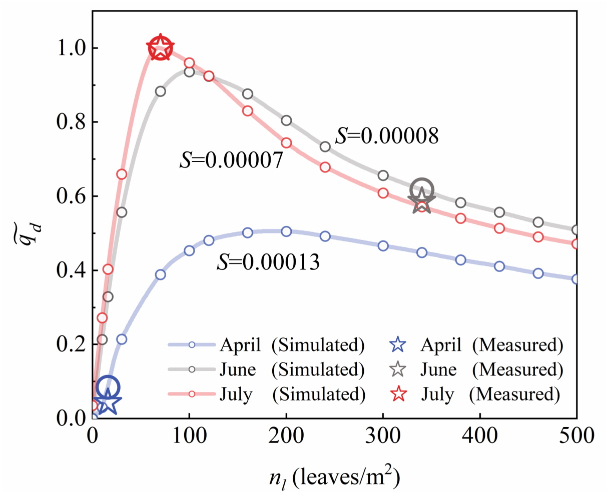

Fig. S5 | Modeling curves for each measurement condition. The large circles represent the predicted sedimentation rate, which correspond to measurement conditions.

References

1. Pritchard, D. Rate of Deposition of Fine Sediment from Suspension. *Journal of Hydraulic Engineering* **132**, 533–536 (2006).
2. Fischer, H. B. Longitudinal Dispersion and Turbulent Mixing in Open-Channel Flow. *Annual Review of Fluid Mechanics* **5**, 59–78 (1973).
3. Xu, Y. & Nepf, H. Suspended Sediment Concentration Profile in a Typha Latifolia Canopy. *Water Resources Research* **57**, e2021WR029902 (2021).
4. Lu, S. Q. Experimental study on suspended sediment distribution in flow with rigid vegetation PhD thesis. Hohai University (2008) (in Chinese).
